# DEPRESSIVE SYMPTOMS IN CENTENARIANS DURING THE COVID-19 PANDEMIC: FINDINGS FROM THE SWISS100 STUDY

**DOI:** 10.1093/geroni/igad104.0919

**Published:** 2023-12-21

**Authors:** Carla Gomes da Rocha, Armin von Gunten, Justine Falciola, Kim Uittenhove, Stefano Cavalli, Francois Herrmann, Mike Martin, Daniela Jopp

**Affiliations:** HES-SO Valais-Wallis, Lausanne, Vaud, Switzerland; Lausanne University Hospital, Prilly, Vaud, Switzerland; Geneva University Hospital, Thônex, Geneve, Switzerland; University of Lausanne, Lausanne, Vaud, Switzerland; University of Applied Sciences and Arts of Southern Switzerland, Manno, Ticino, Switzerland; Geneva University Hospitals, Thônex, Geneve, Switzerland; University of Zurich, Zurich, Zurich, Switzerland; University of Lausanne, Lausanne, Vaud, Switzerland

## Abstract

Depressive symptoms (DS) are prevalent among older adults (WHO, 2021). However, data on DS appear to be scarce in centenarians. In addition, DS may have been aggravated by the COVID-19 pandemic, especially in centenarians who may have been more susceptible to the negative consequences of pandemic restrictions. Thus, as part of the SWISS100 study (Jopp et al., 2023), we measured DS in centenarians living in Switzerland during the COVID-19 pandemic. Randomly selected centenarians from across the country and their proxies were invited to participate in a telephone interview between December 2020 and June 2022. The study sample was composed of 171 centenarians, with a mean age of 101.8 (SD=1.7) years; 128 (74.9%) participants were female and 63 (36.8%) lived at home. DS were assessed via five selected items of the Geriatric Depression Scale (Sheikh and Yesavage, 1986). The mean DS score was 1.6 (SD=1.6). Considering a cut-off ≥2 (Brañez-Condorena et al., 2021), 75 (43.9%) centenarians were screened positive for possible depression. Further analysis indicated that women had a higher level of DS, and that nursing home residents were more depressed than community-dwelling centenarians. Thus, our study suggests that almost half of the centenarians may have presented with relevant DS during the pandemic in Switzerland, which is notably higher than in other studies. DS in centenarians should be screened systematically, not less so in the context of a health crisis. Depressive symptomatology in the very old is highly relevant for successful professional care and needs further investigation to develop best practice.

